# Induction of desiccation tolerance in desiccation sensitive *Citrus limon* seeds

**DOI:** 10.1111/jipb.12788

**Published:** 2019-03-28

**Authors:** Alexandre Marques, Harm Nijveen, Charles Somi, Wilco Ligterink, Henk Hilhorst

**Affiliations:** ^1^ Laboratory of Plant Physiology Wageningen University and Research Wageningen The Netherlands; ^2^ Bioinformatics Group Wageningen University and Research Wageningen The Netherlands

## Abstract

Many economically important perennial species bear recalcitrant seeds, including tea, coffee, cocoa, mango, citrus, rubber, oil palm and coconut. Orthodox seeds can be dried almost completely without losing viability, but so‐called recalcitrant seeds have a very limited storage life and die upon drying below a higher critical moisture content than orthodox seeds. As a result, the development of long‐term storage methods for recalcitrant seeds is compromised. Lowering this critical moisture content would be very valuable since dry seed storage is the safest, most convenient and cheapest method for conserving plant genetic resources. Therefore, we have attempted to induce desiccation tolerance (DT) in the desiccation sensitive seeds of *Citrus limon*. We show that DT can be induced by paclobutrazol (an inhibitor of gibberellin biosynthesis) and we studied its associated transcriptome to delineate the molecular mechanisms underlying this induction of DT. Paclobutrazol not only interfered with gibberellin related gene expression but also caused extensive changes in expression of genes involved in the biosynthesis and signaling of other hormones. Paclobutrazol induced a transcriptomic switch encompassing suppression of biotic‐ and induction of abiotic responses. We hypothesize that this is the main driver of the induction of DT by paclobutrazol in *C. limon* seeds.

**Edited by:** John Harada, University of California, Davis, USA

## INTRODUCTION

Desiccation tolerance (DT) refers to the capacity of an organism to dehydrate to below 10% water on a fresh weight basis (or 0.1 g H_2_O/g dry weight) without accumulation of lethal damage (Alpert 2005). This mechanism was essential for the colonization of terrestrial habitats by the early land plants. Vegetative DT is very common in basal plants and is mostly confined to seeds of spermatophytes (Oliver et al. 2000). However, seed DT has been lost in some plants adapted to environments where conditions are conducive to immediate germination (Baskin and Baskin 2014).

Of the spermatophytes worldwide, 8% display seed desiccation sensitivity (DS; Tweddle et al. 2002). This number can reach up to 50% in tropical evergreen rainforests (Tweddle et al. 2003). The lack of DT places these species on the list of high risk of extinction, as their seeds cannot be stored by conventional means in seed banks. This severely hampers the long‐term conservation of their genetic resources.

Although seeds of many citrus cultivars can be dehydrated safely to 10%–12% moisture content, further dehydration impairs seed viability (Wen et al. 2010). However, there is considerable variability among seed lowest safe moisture content (LSMC) when comparing different studies on DT in *Citrus limon* seeds, resulting in their various categorization by different authors from orthodox (King et al. 1981) and intermediate to recalcitrant (Farnsworth 2000; Hong et al. 2001; Black and Pritchard 2002; Khan et al. 2002; Khan et al. 2003). In our hands *C. limon* seeds are considered desiccation sensitive as they cannot be dried below a water content of 0.1 g water per gram dry weight without losing viability, which defines DT (Bewley et al. 2013).

The phytohormones abscisic acid (ABA) and gibberellins (GAs) are associated with many physiological processes and often work antagonistically. During seed maturation, ABA is responsible for the acquisition of DT in orthodox seeds (Gutierrez et al. 2007). Low levels of ABA or disruption of its signaling pathway may result in desiccation sensitivity (Ooms et al. 1993). GA is associated with the breaking of dormancy and induction of germination (Hilhorst and Karssen 1992). Furthermore, high levels of GA have been associated with vivipary and, consequently, DS (White et al. 2000).

The acquisition of DT in orthodox seeds or lack thereof in desiccation sensitive seeds is assumed to be a consequence of a changed balance between these phytohormones (Bewley et al. 2013). The possibility of induction of DT in a desiccation sensitive seed has been demonstrated in *Acer saccharinum* seeds (Beardmore and Whittle 2005). A combination of ABA and tetcyclacis, an inhibitor of ABA catabolism and GA biosynthesis (Rademacher et al. 1987), induced DT in these seeds (Beardmore and Whittle 2005).

Here we report, for the first time, the induction of DT in the desiccation sensitive seeds of a crop by treatment with paclobutrazol (PAC), an inhibitor of GA‐biosynthesis. The growth retardant inhibits GA‐biosynthesis by the inhibition of the oxidation of ent‐kaurene to ent‐kauronoic acid through inactivating cytochrome P450‐dependent oxygenase (Zhu et al. 2004). The induction of DT in *C. limon* seeds and the analysis of the associated transcriptional changes points to a potential mechanism of acquisition of DT in otherwise DS seeds. Furthermore, the possibility of induction of DT in seeds may support breeding, germplasm storage and further commercialization of *C. limon*. In addition, this study may provide useful insights in the development of protocols to induce DT in other DS seeded species.

## RESULTS

### Induction of DT in desiccation sensitive seeds of *Citrus limon*

Seeds of different Citrus species vary widely in their level of DT (Khan et al. 2003). *C. limon* seeds have been considered orthodox, intermediate or recalcitrant by different studies (Khan et al. 2002). In this study, seeds from the *Citrus limon (L.) Burm. f*. cultivar Primofiori, displayed intermediate storage behavior as they showed severe loss of viability when dried to 10% water content on a fresh weight basis (Marques et al. 2018). According to this classification, recalcitrant seeds lose viability when dried below approximately 25% water content on a fresh weight basis.

**Figure 1:**
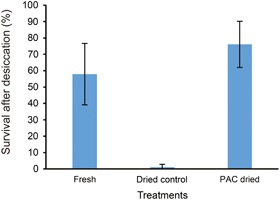
**Total germination of *C. limon* seeds** ‘Fresh’ germination of seeds immediately after removal from the fruits; germination of ‘Dried Control’, seeds after desiccation to 10% water content; ‘PAC Dried’, seeds treated by PAC and dried subsequently to 10% water content. Bars represent the average of four replicates of 25 seeds each. Error bar represents the standard deviation of those four replicates.

With the aim to induce DT in *C. limon* seeds, we first exposed the seeds to different concentrations of ABA (5, 50 and 500 μM). Even at the highest concentration of ABA, high percentages of germination (>90%) were observed so this treatment would not induce DT in these seeds as germinated seeds are desiccation sensitive, even in orthodox seeds. Subsequently, we tested manipulation of GA content. Treatment of whole seeds with 10 mmol/L paclobutrazol (PAC), an inhibitor of gibberellin biosynthesis, for 7 d, restored germination to 80% after desiccation, while non‐treated control seeds showed less than 1% of survival at 10% water content (Figure 1). As expected, fresh seed germination was also inhibited by PAC (data not shown).

These results imply that a treatment with PAC prevented seeds from germinating and protected them against desiccation damage. PAC‐treated dried seeds even displayed a high percentage of germination after 6 months of dry storage. However, after 9 months of dry storage, the survival of PAC‐treated seeds had dropped sharply (Table 1).

**Table 1:** Survival assessment of desiccated PAC‐treated seeds of *C. limon* after storage

Months of dry storage	Survival (%)	Water content (% fresh weight)
0	83 ± 9	10 ± 0.8
6	69 ± 13	7.9 ± 0.5
9	5 ± 3	7.1 ± 0.3

### Transcriptional changes triggered by PAC

To investigate the molecular changes associated with PAC induced DT, we performed a transcriptome analysis on non‐desiccated seeds incubated for 7 d in PAC and in water (control). We used RNA‐seq analysis to identify the genes of which the expression had changed in desiccation tolerant PAC‐treated seeds in comparison to non‐treated desiccation sensitive fresh seeds, using three biological replicates per condition (Figures S1, S2). Since there is no genome sequence available for *C. limon*, we executed a *de novo* assembly of the RNA‐seq reads using the Trinity software (Haas et al. 2013). This resulted in 103,841 predicted ‘genes’ and 179,588 transcripts (Tables S1, 2). Using blastx we found hits with 16,003 *Arabidopsis* genes for 45,425 *C. limon* assembled transcripts.

**Figure 2:**
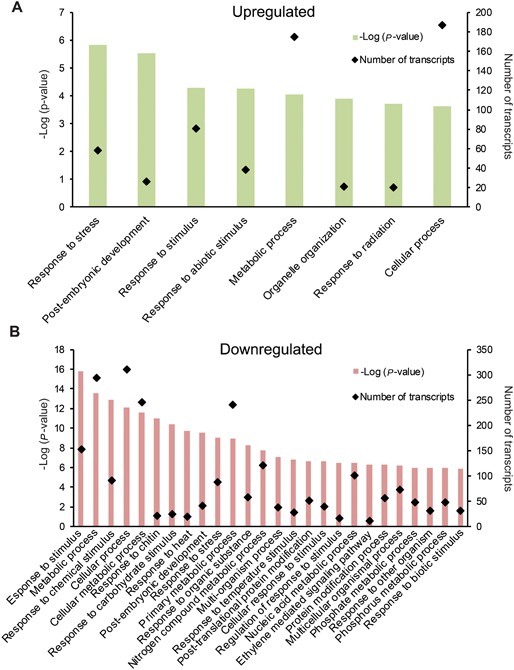
**Over‐representation analysis of the differentially expressed genes after PAC treatment** The gene set analyzed was first filtered through a fold‐change and variance cut‐off based filter (fold change >2 or <‐2 and FDR < 0.05). (**A**) The biological process GOs enriched in the upregulated set. (**B**) The top 26 biological process GOs in the downregulated set based on *P*‐value. The total list of enriched GO categories can be found in Table S4.

Seed samples treated with PAC showed significant upregulation of 613 genes and downregulation of 950 genes in comparison to the water control (fold change > 2 or <‐2 respectively with FDR < 0.05). Based on a GO enrichment analysis of the upregulated set, we found eight biological process (BP) categories significantly enriched (FDR < 0.05), as well as 31 molecular function (MF) and 35 cellular component (CC) categories. The downregulated set showed 98 BP, 20 MF and 17 CC categories (Figure 2). For a complete list of differentially regulated genes and their KEGG and GO enriched categories, see Table S3.

The downregulated set contained more genes and was more diverse than the upregulated set, as evidenced by the much larger number of GO categories. This likely represents the general growth inhibitory action of the PAC treatment. Apparently, the suppression of GA‐biosynthesis has multiple effects across diverse pathways.

Furthermore, a cellular compartmentalization switch was observed in the cellular component GO enrichment analysis. The mitochondrial transcriptome appeared largely suppressed, whereas plastid genes were induced (Table S5). The reduction of mitochondrial gene expression likely reflects the suppression of energy metabolism and preparation for quiescence. The upregulation of genes located in the plastids is not photosynthesis related as these seeds are not green. Thus, this feature may rather be associated with reserve remobilization, particularly that of lipids. We detected the upregulation of plastid located genes such as *FATTY ACID DESATURASE 6* (*FAD6*), *ACETYL CO‐ENZYME A CARBOXYLASE BIOTIN CARBOXYLASE SUBUNIT* (*CAC2*), *ASPARTATE AMINOTRANSFERASE 3* (*ASP3*), *CYTIDINEDIPHOSPHATE DIACYLGLYCEROL SYNTHASE 4* (*CDS4*), *LIPOYL SYNTHASE 1* (*LIP1*) and *PLASTID LIPASE1* (*PLIP1*).

Furthermore, the heat shock transcription factors *HSFA2*, *HSFA4A* and *HSFA6B* were upregulated. Additionally, we found upregulation of the genes encoding the heat shock proteins *HEAT SHOCK COGNATE PROTEIN 70‐1* (*HSC70‐1*), *HEAT SHOCK PROTEIN 60* (*HSP60*) and *HEAT SHOCK PROTEIN 101* (*HSP101*). We did not observe an extensive upregulation of genes related to protective mechanisms against desiccation, besides the antioxidant enzyme *CATALASE 2* (*CAT2*). There were 43 LEAs, commonly associated with the acquisition of DT (Hundertmark and Hincha 2008), identified in the transcriptome of both control and PAC treated seeds. However, there was no significant upregulation of LEA transcripts. We identified only three LEAs, belonging to Group 2 (Hundertmark and Hincha 2008), which were downregulated upon PAC treatment (Table S6).

### PAC promotes abiotic and suppresses biotic responses in *C. limon* seeds

The upregulated genes were enriched with the GO categories *response to abiotic stress* and *response to radiation* (Figure 2A). These categories contain genes responsible for DNA repair and protection. DNA damage is one of the main problems related to desiccation sensitivity, as was demonstrated in various tolerant and intolerant seeds and other organisms (Mattimore and Battista 1996; Boubriak et al. 1997; Boubriak et al. 2000; Faria et al. 2005).

Next to *CAT2*, in the upregulated gene set we found the abiotic stress‐associated genes *EARLY RESPONSIVE TO DEHYDRATION 15* (*ERD15*) and the previously mentioned heat shock transcription factors and proteins.

**Figure 3:**
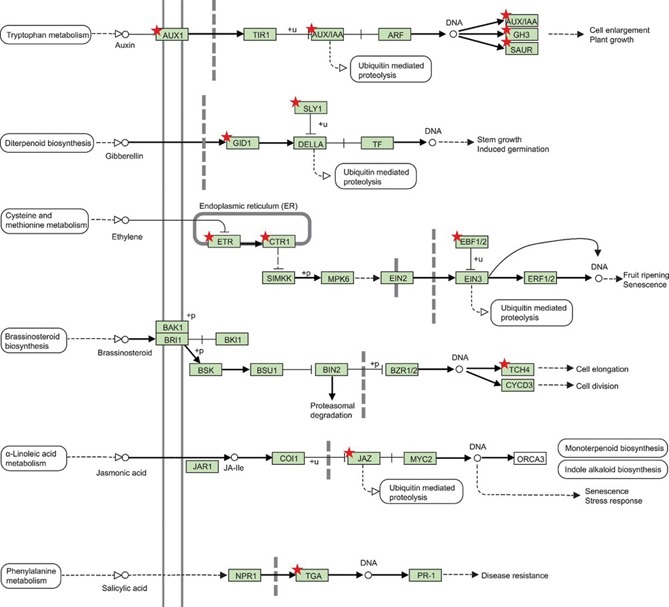
**KEGG enrichment analysis (DAVID) of the genes downregulated upon PAC treatment (fold change < −2 and FDR < 0.05)** The model depicts the signaling pathway of multiple plant hormones. The red stars indicate downregulated genes upon PAC treatment of *C. limon* seeds.

Genes related to biotic hormonal pathways were downregulated (Figure 3). Moreover, there was an extensive downregulation of genes involved in biotic responses, such as those in the categories *response to chitin* and *response to biotic stimulus* (Figure 2B).

### Hormonal interactions underlying responses to PAC treatment

PAC inhibits the monooxygenases involved in the oxidation of *ent*‐kaurene to *ent*‐kaurenoic acid and therefore reduces the plant's ability to synthesize active GAs (Rademacher 1989, 1991). GAs are an absolute requirement for germination and inhibition of *ent*‐kaurene oxidase by PAC prevents germination (Jacobsen and Olszewski 1993).

Seed germination is regulated by multiple hormones, such as GA, ABA, ethylene, and brassinosteroids (Kucera et al. 2005). The GA‐responsive genes include those responsible for synthesis, transport, and signaling of other hormones, suggesting a crosstalk between GA and other hormones (Ogawa 2003). Here multiple hormonal pathways were downregulated by the PAC treatment of *C. limon* seeds (Figure 3).

We performed a KEGG enrichment analysis of the downregulated gene set and found a significant enrichment of the category *plant hormone signal transduction* (Table S4). Among them were genes involved in the signaling of auxin, gibberellin, ethylene, brassinosteroids, jasmonic and salicylic acid (Figure 3). The downregulated genes involved in auxin signaling were *AUXIN RESISTANT 1* (*AUX1*), *INDOLEACETIC ACID‐INDUCED PROTEIN 16* (*IAA16*), *AUXIN‐RESPONSIVE GH3 FAMILY PROTEIN (GH3.1), SMALL AUXIN UPREGULATED RNA 32* (*SAUR32*), in gibberellin signaling *GIBBERELLIN 2‐BETA‐DIOXYGENASE 2* (*GA2OX2*), *GA INSENSITIVE DWARF1B* (*GID1B*) and *GA INSENSITIVE DWARF2* (*GID2*), in ethylene signaling *CONSTITUTIVE TRIPLE RESPONSE 1* (*CTR1*), *ETHYLENE RESPONSE 2* (*ETR2*) and *EIN3‐BINDING F BOX PROTEIN 1* (*EBF1*), in brassinosteroid signaling *TOUCH 4* (*TCH4*), in jasmonic acid signaling *JASMONATE‐ZIM‐DOMAIN PROTEIN 1,2* and *3* (*JAZ1, JAZ2* and *JAZ3*) and in salicylic acid signaling *TGACG (TGA) MOTIF‐BINDING PROTEIN 10* (*TGA10*).

Conversely, we observed the upregulation of transcripts involved in ABA signaling, such as *MOTHER OF FT AND TFL1* (*MFT*), *MEDIATOR OF ABAregulaTED DORMANCY 1* (*MARD1*) and *G‐BOX BINDING FACTOR 3* (*GBF3*). Interestingly these genes are also involved in the control of seed maturation and germination (Zhang et al. 2011; Belmonte et al. 2013; Arana et al. 2014).

### Signaling pathways involved in the control of germination and seed desiccation sensitivity in *C. limon seeds*

Seed desiccation sensitivity has been associated with the lack of developmental arrest. Thus, we took a closer look at the differentially regulated genes involved in the promotion or inhibition of germination and, possibly, with acquisition of DT in *C. limon* seeds upon PAC treatment.

In addition to *MFT*, *MARDI1* and *GBF3*, there was downregulation of PhyE and *SCL3* which are transcripts involved in the stimulation of seed germination through the GA pathway (Zhang et al. 2011; Arana et al. 2014). *REVEILLE 1* (RVE1) is a promoter of primary seed dormancy in *Arabidopsis*; its overexpression can complement the non‐dormant phenotype of the *delay of germination 1* (*dog1*) mutant. It was upregulated in PAC treated *C. limon* seeds. RVE1 has also been shown to suppress GA‐biosynthesis by binding directly to the promoter of *GIBBERELLIN‐3‐OXIDASE 2* (*GA3ox2*) (Jiang et al. 2016).

Furthermore, we performed an enrichment analysis to find enriched consensus transcription factor (TF) binding sites in the set of differentially regulated genes upon treatment with PAC (Jin et al. 2017). The upregulated genes showed significant enrichment (*P* < 0.001) of motifs for 30 TFs (Table 2). Of these TFs, three were identified in the upregulated gene set, i.e., *RVE1, RVE7* and *GBF3*.

**Table 2:** Transcription factors that have over‐represented targets on the PAC‐treated upregulated genes

TF	Gene ID	*p*‐value	Query	Background
**AT5G17300**	**REVEILLE 1 (RVE1)**	**7.45E‐07**	**31**	**711**
AT3G09600	REVEILLE 8 (RVE8)	2.77E‐06	30	720
AT2G01930	BASIC PENTACYSTEINE1 (BPC1)	3.17E‐06	195	8,742
AT3G23210	BASIC HELIX‐LOOP‐HELIX 34 (bHLH34)	3.54E‐06	56	1,779
AT4G38910	BASIC PENTACYSTEINE 5 (BPC5)	4.23E‐06	169	7,371
**AT1G18330**	**REVEILLE 7 (RVE7)**	**1.37E‐05**	**28**	**705**
AT3G10113	Homeodomain‐like superfamily protein	1.65E‐05	28	712
AT5G52660	REVEILLE 6 (RVE6)	3.57E‐05	28	743
AT3G10800	(BZIP28)	7.39E‐05	54	1,888
AT2G36270	ABA INSENSITIVE 5 (ABI5)	9.37E‐05	56	1,997
AT5G42520	BASIC PENTACYSTEINE 6 (BPC6)	1.41E‐04	160	7,334
**AT2G46270**	**G‐BOX BINDING FACTOR 3 (GBF3)**	**1.79E‐04**	**51**	**1,816**
AT3G56850	ABA‐RESPONSIVE ELEMENT BINDING PROTEIN 3 (AREB3)	1.82E‐04	55	2,002
AT1G32150	TRANSCRIPTION FACTOR 68 (bZIP68)	1.93E‐04	54	1,960
AT1G45249	ABSCISIC ACID RESPONSIVE ELEMENTS‐BINDING FACTOR 2 (ABF2)	1.99E‐04	55	2,009
AT4G01280	REVEILLE 5 (RVE5)	2.11E‐04	23	625
AT3G20840	PLETHORA 1 (PLT1)	2.12E‐04	17	402
AT5G08130	(BIM1)	2.62E‐04	30	917
AT4G00250	(ATSTKL2)	2.85E‐04	17	412
AT2G35530	BASIC REGION/LEUCINE ZIPPER TRANSCRIPTION FACTOR 16 (bZIP16)	2.86E‐04	53	1,945
AT5G61270	PHYTOCHROME‐INTERACTING FACTOR7 (PIF7)	3.39E‐04	41	1,409
AT5G02840	LHY/CCA1‐LIKE 1 (LCL1)	5.31E‐04	21	587
AT1G10120	CRY2‐INTERACTING BHLH 4 (CIB4)	5.48E‐04	35	1,175
AT5G67450	ZINC‐FINGER PROTEIN 1 (ZF1)	5.95E‐04	37	1,269
AT5G61620	myb‐like transcription factor family protein	7.41E‐04	17	447

In bold are the transcription factors that were identified in the RNA‐seq analysis as upregulated.

**Figure 4:**
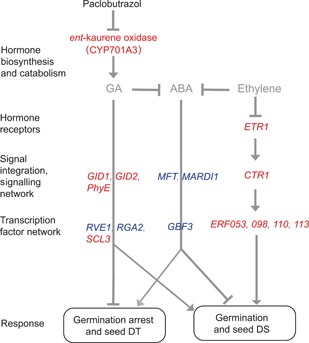
**Schematic representation of the interactions between the gibberellin (GA), abscisic acid (ABA) and ethylene signaling pathways in the regulation of the arrest of germination and DT in seeds of *C. limon* treated with paclobutrazol (PAC)** Note that CYP701A3 is the protein. Upregulated genes are marked green and downregulated ones red. The model is mainly based on *Arabidopsis* hormone mutant analyses adapted from Kucera et al. (2005). The positions of some components are speculative and details are discussed in the text.

All relevant transcriptional changes identified in the present study involving hormonal signaling and germination control are shown in Figure 4.

## DISCUSSION

### Induction of DT in desiccation sensitive seeds of *Citrus limon*

The storage of desiccated seeds is important to ensure long‐term conservation of a broad genetic diversity. In the present study, *C. limon* seeds showed a DS phenotype and, hence, cannot be stored for prolonged periods.

It is challenging to determine the cause of the wide variation of seed desiccation sensitivity across the different *C. limon* studies. Here we studied a desiccation sensitive phenotype of *C. limon* the seeds of which completely lost viability at 10% water content. Possible sources of this variation can be different agronomical practices, adaptation to different environments or varying genetic backgrounds. One of the main breeding targets for citrus species is to lower seed numbers and produce seedless fruits (Varoquaux et al. 2000). This breeding throughout the long *Citrus* domestication history has contributed to the transition to apomixis and reduction of seed numbers (Wang et al. 2017). These domestication events towards loss of seeds possibly contributed to the reduction of their quality, influencing traits such as longevity and/or DT. Citrus species originate in southeast Asia and have been cultivated for thousands of years (Velasco and Licciardello 2014).

Variability in seed dehydration tolerance is also apparent in some other genera, e.g. *Araucaria*, *Dipterocarpus* and *Coffea* (Tompsett 1984; Tompsett 1987; Ellis et al. 1991). This interspecific dehydration tolerance variability may be the result of adaptation to different environments. However, it is also possible that both the adaptation to different environments and breeding contributed to the seed DT variability observed in *Citrus*.

Here we report, for the first time, induction of DT in a DS‐seeded crop. The arrest of germination by inhibiting gibberellin synthesis in recalcitrant seeds was first described in *Hopea odorata* (Garello and Le Page‐Degivry 1995). However, the induction of DT in a DS seed has so far only been described for *Acer saccharinum* seeds (Beardmore and Whittle 2005). Similar to *C. limon*, these seeds are also insensitive to ABA. Application of ABA alone resulted in 0% survival after drying, but ABA combined with tetcyclacis, which inhibited both ABA catabolism and GA synthesis (Rademacher et al. 1987), induced DT in *A. saccharinum* seeds. We successfully induced DT in *C. limon* seeds with the GA biosynthesis inhibitor PAC.

*A. saccharinum* seeds displayed loss of viability when desiccated to water contents below 30%, characterizing recalcitrant behavior (Becwar et al. 1983). In this study, *C. limon* seeds lost viability when dried to 10% water content based on fresh weight. Although we did not evaluate the water content when these seeds started to lose viability, other studies have shown survival of *C. limon* seeds to water contents below 10%, but always displaying some degree of desiccation sensitivity (King et al. 1981; Soetisna et al. 1985; Hong et al. 2001). Taken together, it suggests that *C. limon* seeds have an intermediate seed storage behavior with a variable LSMC. Based on this, we expect that *A. saccharinum* seeds are more sensitive to desiccation than *C. limon* and, thus, induction of DT in *A. saccharinum* is likely to be more complex. The seeds of *A. saccharinum* required a treatment which inhibited both GA‐biosynthesis and ABA catabolism while *C. limon* seeds only required the inhibition of GA‐biosynthesis to acquire DT.

The viability of PAC‐treated seeds remained high until 6 months after desiccation but dropped quickly after 9 months. The viability of these seeds dropped concomitantly with a small reduction of their water content to 7.9% (Table 1). Therefore, we cannot conclude if this loss of viability is due to ageing related deterioration, the reduction in water content or both.

DT in orthodox seeds has been shown to be associated with multiple genes that are most likely additive to warrant good seed longevity (Marques et al. 2018). The mis‐expression of some of these genes is likely to result in reduced longevity. It is likely that the DT induced in PAC treated *C. limon* seeds was not as ‘complete’ as the DT observed in orthodox seeds. The analysis of the transcriptome of PAC treated seeds can shed light on which genes are responsible for the desiccation induction in *C. limon* seeds and their limited longevity.

### Overall transcriptional changes triggered by paclobutrazol

GAs and ABA are important plant growth regulators, acting antagonistically to control many plant developmental processes, including root and stem elongation, rosette expansion, floral induction, anther development and seed germination (Yamaguchi 2008). GA levels are normally decreased and those of ABA increased at later stages of orthodox seed maturation and abnormal high levels of GA can cause precocious germination (White et al. 2000).

Current consensus suggests that seed desiccation sensitivity occurs when seeds fail to undergo the final stages of maturation during seed development (Farrant and Moore 2011). During maturation, a number of key events occur, including the synthesis and deposition of stored reserves (storage proteins, carbohydrates and lipids) and LEA proteins (Verdier et al. 2013).

Beardmore and Whittle (2005) suggested that, by treatment with ABA, desiccation sensitive embryonic axes of *A. saccharinum* probably underwent a process of seed maturation as in orthodox seeds. They associated the induction of DT with the accumulation of storage proteins and dehydrins.

However, upon PAC treatment of *C. limon* seeds, we did not observe significant upregulation of maturation and desiccation related genes, such as those encoding for seed storage proteins and LEAs, except for HSPs and catalase. Three LEAs, belonging to group 2, were downregulated upon PAC treatment. Furthermore, this group of LEAs has not been associated with the acquisition of seed DT and its classification as a group of LEA proteins is controversial (Hundertmark and Hincha 2008; Chatelain et al. 2012). It is possible that PAC induced DT in *C. limon* seeds by the regulation of a few essential protective elements whereas most of these were already present in control seeds, e.g. genes encoding 43 LEAs were present in both treatments without significant changes in expression. Furthermore, we observed that the transcriptomes of both control and PAC treated *C. limon* seeds resembled that of *Arabidopsis* seeds imbibed for 24 h in ABA, which have not germinated yet and are still desiccation tolerant (Waese and Provart, 2017).

During the development of orthodox seeds the reduction of metabolism, particularly the reduction of respiration rate, coincides with their survival after desiccation (Leprince et al. 1999; Pammenter and Berjak 1999). We assume that high GA and low ABA levels in DS seeds are responsible for the maintenance of active metabolism and the skipping of maturation drying. Thus, the inhibition of GA synthesis in *C. limon* seeds counteracted these processes and thus arrested the germination program, suppressed metabolic activity and allowed successful desiccation.

### Does PAC treatment rescue *C. limon* seed DT by upregulating abiotic and suppressing biotic responsive genes?

The recalcitrant seeds of *Garcinia mangostana* accumulate secondary metabolites particularly related to phenylpropanoid pathway. The authors argued that these metabolites are likely responsible for enhancing these seeds biotic defense (Mazlan et al. 2018). We found in PAC treated *C. limon* seeds the downregulation of genes involved in hormonal pathways of biotic stress responses, such as jasmonic acid (*ATJAZ1, JAZ2* and *JAZ3*) and salicylic acid (*TGA10*). We also observed the GO enrichment of biotic response categories among the downregulated genes set, such as *response to chitin* and *response to biotic stimulus*.

Chitin has been demonstrated to be an elicitor of fungal infection responses in several plants, including Citrus (Gallão et al. 2007; Eckardt 2008; Boller and Felix 2009). Furthermore, the downregulation of biotic responses, including *response to chitin*, was also demonstrated for the re‐establishment of DT in germinated seeds of *Arabidopsis thaliana* upon ABA treatment (Costa et al. 2015). The protection towards biotic agents is important for imbibed seeds, especially for those seeds shed in ripened fleshy fruits, like most recalcitrant seeds in the tropical rainforests (Chin 1977), that are more prone to fungal attack. Chitin triggers lignin deposition as a strategic response to combat fungal infection (El Hadrami et al. 2010). However, lignin hardens the cell walls while DT requires flexible and foldable cells (Moore et al. 2008). It has been demonstrated that recalcitrant seeds show cell wall damage when desiccated (Chandel et al. 1995; Moore et al. 2008). We hypothesize that the downregulation of biotic responses is not only an isolated consequence of the metabolic arrest caused by PAC but an essential response that prevents the lignification of the cell walls of *C. limon* seeds and thus make these flexible, allowing their folding and preventing damage upon seed desiccation.

The inverse relation between the regulation of biotic versus abiotic stresses and associated hormone pathways, as observed in the present work, has also been found in other studies where, for example, ABA contents are correlated with susceptibility to pathogens in diverse plant species (Mauch‐Mani and Mauch 2005). We assume that the downregulation of biotic responses and the upregulation of abiotic responses in PAC treated *C. limon* seeds that leads to DT are interconnected by common regulatory pathways.

We detected the upregulation of the heat shock transcription factors *HSFA2*, *HSFA4A* and *HSFA6B* that are known to regulate HSPs. Heat shock regulated transcription factors and HSPs are an interesting class of upregulated genes as they have been associated with diverse abiotic stresses (Wang et al. 2004). It has been suggested that HSPs act as chaperonins to protect other proteins under various stresses (Wang et al. 2004). This chaperonin protective role of HSPs has been shown to provide vegetative abiotic stress tolerance, such as tolerance to heat, salt and oxidative stress in transgenic *Arabidopsis* studies (Chauhan et al. 2013; Pérez‐Salamó et al. 2014).

Small heat shock proteins (*sHSPs*) have been associated with the acquisition of DT (Wehmeyer et al. 1996; Wehmeyer and Vierling 2000). However, we did not find the upregulation of sHSPs but only of HSPs: *HEAT SHOCK COGNATE PROTEIN 70‐1* (*HSC70‐1*), *HEAT SHOCK PROTEIN 60* (*HSP60*) and *HEAT SHOCK PROTEIN 101* (*HSP101*). *HSC70‐1* regulates development and abiotic stress responses in *A. thaliana* (Leng et al. 2017), *HSP60* has been implicated in heat shock responses in *Arabidopsis* (Rikhvanov et al. 2007) and *HSP101* is the most abundant heat stress protein in seeds. It was not detectable in the *A. thaliana* DS‐seeded mutant, *abi3‐6* (Kotak et al. 2007).

### Transcriptional changes associated with hormonal balance and germination control

We expected that the inhibition of gibberellin biosynthesis by PAC, which inhibits the enzyme *ent*‐kaurene oxidase (Dalziel and Lawrence 1984), would downregulate the downstream genes involved in gibberellin biosynthesis and signaling. Indeed, we observed downregulation of *GA2OX2,* the gene encoding for the rate limiting enzyme GA2 oxidase in gibberellin biosynthesis, as well as *GID1B* and *GID2* which are members of the GID GA‐receptor family. The knock‐outs of *GID1* and *GID2* have been shown to suppress the germination of *Arabidopsis* seeds (Hauvermale et al. 2015; Nelson and Steber 2017).

The balance between ABA and GA has been demonstrated to be decisive for the regulation of many physiological processes (Olszewski et al. 2002; Razem et al. 2006). Of the ABA biosynthesis genes that responded to the PAC treatment, we found that the transcript coding for *NCED6* was upregulated but the transcripts coding for the downstream enzyme molybdenum cofactor sulfurase (*ABA3*) were downregulated. Thus, we expect that the ABA levels are increased as a result of the PAC treatment, although no ABA measurements were performed in this study. Genes that have been shown to be induced by ABA were also upregulated by PAC, such as *GBF3*, *GPCR‐type G protein 1*, *MFT* and *MARD1*.

The upregulation of *MFT*, *MARD1* and *GBF3* has been associated with the arrest of germination and these genes are possibly involved in the acquisition of DT in *C. limon* seeds upon PAC treatment (Figure 4). The transcription factor *G‐box binding factor 3* (*GBF3*) is involved in the water stress response during the desiccation phase of *A. thaliana* seed formation (Wohlbach et al. 2008). Furthermore, *MFT* responds to both ABA and GA signaling to regulate seed germination in *A. thaliana* (Xi et al. 2010) and *MARD1* plays a role in the arrest of germination through ABA‐mediated dormancy (He and Gan 2004). The lack of developmental arrest has been associated with seed desiccation sensitivity in the *A. thaliana* mutants *fus3‐8*, *lec1‐3* and *abi3‐5* (Raz et al. 2001).

*GBF3* is involved in an abscisic acid‐dependent signal transduction pathway under drought‐ and high‐salinity conditions (Uno et al. 2000), as well as in the regulation of seed maturation (Belmonte et al. 2013). Together with other bZIP transcription factors, such as *ABI5* and *EEL*, *GBF3* regulates seed maturation and acquisition of DT (Bensmihen et al. 2002; González‐Morales et al. 2016). Several upregulated genes are predicted to be targets of *GBF3*. It suggests that *GBF3* is an important regulator of the transcriptional changes occurring in PAC treated *C. limon* seeds and, possibly, of the acquisition of DT in these seeds.

We also observed the downregulation of other hormonal pathway‐associated genes, such as ethylene, which is also a promoter of germination (Linkies and Leubner‐Metzger 2012). The mechanism of this downregulation is not known. Extensive downregulation of ethylene biosynthesis and ethylene responsive genes occurred in PAC treated seeds. We assume that the downregulation of the ethylene pathway is also likely to be associated with arrest of germination of the *C. limon* seeds upon PAC treatment. *Arabidopsis* mutants insensitive to ethylene, such as *etr1* and *ein2*, are hypersensitive to ABA during germination (Beaudoin et al. 2000; Ghassemian et al. 2000). Endogenous ABA levels are elevated in *etr1* compared with those in the wild type, suggesting that ethylene signaling negatively affects ABA levels, and as a result, *etr1* seeds are more dormant (Chiwocha et al. 2005). Taken together, the transcriptomic changes, resulting from the PAC treatment in *C. limon* seeds, are predominantly related to the suppression of metabolism as a consequence of the downregulation of multiple hormonal pathways.

## MATERIALS AND METHODS

### Induction of DT

*Citrus limon* (L.) *Burm. f*. (variety Primofiore, cultivated in Spain) seeds were collected from ripe fruits, rinsed with water and sterilized in 10% (v/v) bleach for 10 min. For the induction of DT, seeds were incubated for 7 d in MS medium containing 1% of agar, 5% of sucrose and 10 mmol/L (2RS,3RS)‐1‐(4‐chlorophenyl)‐4,4‐dimethyl‐2‐(1,2,4‐triazol‐1‐yl)pentan‐3‐ol (paclobutrazol, PAC) (Sigma Aldrich).

A preliminary experiment was performed that used the same protocol as described above but using abscisic acid (ABA) (Sigma Aldrich) instead of PAC. We used different concentrations of ABA (5 μM, 50 μM and 500 μM) but they failed to arrest germination and induce DT in *C. limon* seeds. Also, fresh seed germination could not be inhibited by ABA. All the following experiments performed in this study were based on 10 mmol/L PAC treatments.

All drying treatments were performed by placing the seeds in a cabinet for 7 d at 30% relative humidity, with forced ventilation, at 22°C in the dark, resulting in a water content of 10 ± 0.8%. Water content of the seeds was determined by oven drying. After weighing seeds were transferred to an oven set at 105°C. After 16 h at this temperature the seed were weighed again and the weight difference divided by the initial weight resulted in the water content on a fresh weight basis.

Seed survival was assessed by placing the desiccated seeds on germination paper soaked with 50 mL of a 100 μM GA solution (GA_4+7_; Berelex, ICI), at 30°C in the dark. Germination and fully developed seedlings were scored after one month.

### Total RNA isolation

Total RNA was extracted from three independent replicates of non‐desiccated seeds after 7 d in PAC or water (control) according to the hot borate protocol modified from Wan and Wilkins (1994). We did not use desiccated seeds because such samples would always include a proportion of dead seeds or tissues. Three replicates of 25 seeds each for each treatment were homogenized and mixed with 800 μL of extraction buffer (0.2 M Na borate decahydrate (Borax), 30 mmol/L EGTA, 1% SDS, 1% sodium deoxy‐cholate (Na‐DOC)) containing 1.6 mg DTT and 48 mg PVP40 which had been heated to 80°C. 1 mg proteinase K was added to this suspension and incubated for 15 min at 42°C. After adding 64 μL of 2 M KCl, the samples were incubated on ice for 30 min and subsequently centrifuged for 20 min at 12,000 g. 270 µL of ice‐cold 8 M LiCl was added to the supernatant in a final concentration of 2 M and the tubes were incubated overnight on ice. After centrifugation for 20 min at 12,000 g at 4°C, the pellets were washed with 750 μL ice‐cold 2 M LiCl. The samples were centrifuged for 10 min at 10,000 g at 4°C and the pellets were re‐suspended in 100 μL DEPC treated water. The samples were cleaned with phenol chloroform and treated with DNAse (RQ1 DNase, Promega) according to the manufacturers protocol. The RNA quality and concentration were assessed by agarose gel electrophoresis and UV spectrophotometry.

### RNA‐seq analysis, assembly and analysis of differential expression

Messenger RNA (mRNA) was isolated from total RNA using mRNA enrichment polyA capture technology (Illumina Incorporated, San Diego, CA, USA) followed by library preparation using random hexamer priming. RNA‐seq was performed strand specific with paired‐end 125 nt reads on an Illumina Hiseq 2500. The reads were quality trimmed using Trimmomatic (Bolger et al. 2014). The transcriptome was *de novo* assembled using Trinity version 2.1.1 (Grabherr et al. 2011). Blastx (Altschul et al. 1997) with an *E*‐value cut‐off of 1e^−5^ was used to find homologous *Arabidopsis* genes (TAIR10 [Lamesch et al. 2011]) for the assembled genes. Expression was quantified using the Kallisto software tool with the *de novo* assembled transcriptome (Bray et al. 2016) and then analyzed using Sleuth (Pimentel et al. 2017). All subsequent analyses were performed based on the *de novo* assembly.

The RNA‐seq assembly is available at the National Centre for Biotechnology Information (NCBI) as Bioproject ID SRP111791.

### Gene enrichment analysis

Gene Ontology enrichment was performed on significantly up‐ and downregulated gene sets using *Arabidopsis thaliana* orthologs and its genome as reference. We used AgriGo and Revigo to summarize the results (Du et al. 2010; Supek et al. 2011). KEGG enrichment pathway analysis was done with DAVID (Dennis et al. 2003).

## AUTHOR CONTRIBUTIONS

A.M. designed the experiment. A.M. and C.S. performed most of the research. H.N. carried out the RNAseq analysis. W.L. and H.H. supervised the study and revised the manuscript. All authors read and approved the contents of this paper.

## Supporting information

Additional Supporting Information may be found online in the supporting information tab for this article: http://onlinelibrary.wiley.com/doi/10.1111/jipb.12788/suppinfo

**Tables S1, S2 and Figure S1.** Sequencing reads and statistics of RNA‐seq mapping and PCA and dendrogram of Control (C1, C2 and C3) and PAC treated samples (P1, P2 and P3)
**Figure S2.** PCA of Control (C1, C2 and C3) and PAC treated samples (P1, P2 and P3)

**Table S3.** Differentially regulated genes

**Table S4.** KEGG and GO enriched categories of the gene list of Table 2

**Table S5.** Up‐ and down regulated mitochondrial and plastid GO‐categories

**Table S6.** Differentially expressed LEA genes
