# Supplementary material for: Induction of desiccation tolerance in desiccation sensitive Citrus limon seeds
Source: J Integr Plant Biol. 2019 Mar 28;61(5):624–38. doi: 10.1111/jipb.12788 (PMC6593971; doi:10.1111/jipb.12788)
Supplement: Supplementary file 1 — Tables S1, S2 and Figure S1. Sequencing reads and statistics of RNA‐seq mapping and PCA and dendrogram of Control (C1, C2 and C3) and PAC treated samples (P1, P2 and P3) Figure S2. PCA of Control (C1, C2 and C3) and PAC treated samples (P1, P2 and P3) [file JIPB-61-624-s001.docx]

Induction of desiccation tolerance in desiccation sensitive *Citrus limon* seeds

Alexandre Marques^1^, Harm Nijveen^1, 2^, Charles Somi^1^, Wilco Ligterink^1^ and Henk Hilhorst^1^*

# SUPPORTING INFORMATION

**Tables S1, S2 and Figure S1.** Sequencing reads and statistics of RNA-seq mapping and PCA and dendrogram of Control (C1, C2 and C3) and PAC treated samples (P1, P2 and P3)

**Figure S2.** PCA of Control (C1, C2 and C3) and PAC treated samples (P1, P2 and P3)

**Table S3.** Differentially regulated genes.

**Table S4.** KEGG and GO enriched categories of the gene list of Table 1 (Table 2).

**Table S5.** Up- and down regulated mitochondrial and plastid GO-categories

**Table S6.** Differentially expressed LEA genes

# Supplementary Material

**Table S1.** Sequencing reads

| **Sample** | **Reads mapped** | **Reads processed** | **Fraction mapped** |
| --- | --- | --- | --- |
| Control-1 | 15,587,103 | 17,364,908 | 0.8976 |
| Control-2 | 19,938,139 | 22,238,751 | 0.8965 |
| Control-3 | 15,329,299 | 16,657,259 | 0.9203 |
| Paclobutrazol-1 | 16,376,057 | 18,365,376 | 0.8917 |
| Paclobutrazol-2 | 16,870,180 | 18,835,280 | 0.8957 |
| Paclobutrazol-3 | 12,865,185 | 14,398,974 | 0.8935 |

# RNA-seq assembly

**Table S2**. Statistics for RNA-seq mapping

| **Counts of transcripts, GC content.** |
| --- |
| Total trinity 'genes': 103,841 |
| Total trinity transcripts: 179,588 |
| Percent GC: 39.66 |
|  |
| **Statistics based on all transcript contigs** |
| Contig N10: 4,160 |
| Contig N20: 3,277 |
| Contig N30: 2,740 |
| Contig N40: 2,309 |
| Contig N50: 1,933 |
| Median contig length: 612 |
| Average contig: 1,079.95 |
| Total assembled bases: 193,945,868 |
|  |
| **Statistics based on only longest isoform per 'GENE'** |
| Contig N10: 3,984 |
| Contig N20: 2,988 |
| Contig N30: 2,338 |
| Contig N40: 1,765 |
| Contig N50: 1,213 |
| Median contig length: 375 |
| Average contig: 713.71 |
| Total assembled bases: 74,112,540 |


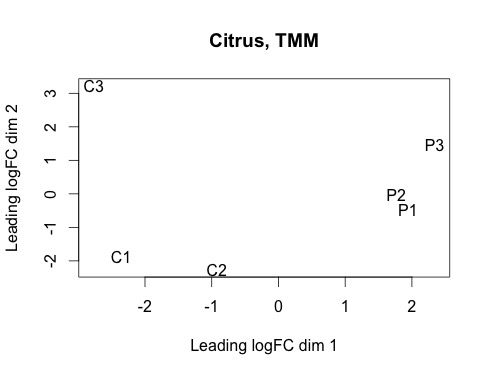


**Figure S1.** PCA of Control (C1, C2 and C3) and PAC treated samples (P1, P2 and P3)


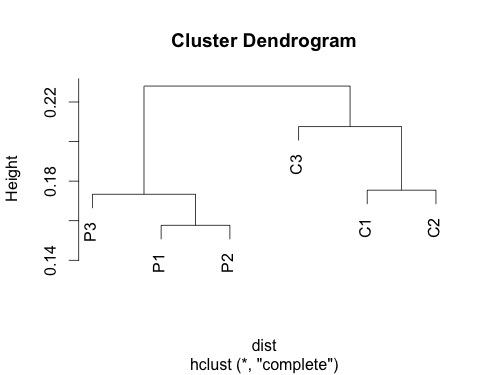


**Figure S2.** PCA of Control (C1, C2 and C3) and PAC treated samples (P1, P2 and P3)
