# Supplementary material for: Induction of desiccation tolerance in desiccation sensitive Citrus limon seeds
Source: J Integr Plant Biol. 2019 Mar 28;61(5):624–38. doi: 10.1111/jipb.12788 (PMC6593971; doi:10.1111/jipb.12788)
Supplement: Supplementary file 4 — Table S5. Up‐ and down regulated mitochondrial and plastid GO‐categories [file JIPB-61-624-s004.docx]

| **Up-regulated GO categories** | |  |  |  |  |  |
| --- | --- | --- | --- | --- | --- | --- |
| **GO_acc** | **Term** | **queryitem** | **querytotal** | **bgitem** | **p-value** | **FDR** |
| GO:0009536 | plastid | 94 | 485 | 2965 | 8E-16 | 2.5E-13 |
| GO:0009507 | chloroplast | 85 | 485 | 2740 | 8.3E-14 | 1.3E-11 |
| GO:0044435 | plastid part | 36 | 485 | 867 | 2E-09 | 5.7E-08 |
| GO:0044434 | chloroplast part | 32 | 485 | 746 | 7.9E-09 | 1.7E-07 |
| GO:0009534 | chloroplast thylakoid | 14 | 485 | 290 | 0.00004 | 0.00069 |
| GO:0031976 | plastid thylakoid | 14 | 485 | 293 | 0.000044 | 0.00072 |
| GO:0009579 | thylakoid | 15 | 485 | 376 | 0.00017 | 0.0023 |
| GO:0034357 | photosynthetic membrane | 11 | 485 | 273 | 0.0011 | 0.012 |
| GO:0009535 | chloroplast thylakoid membrane | 10 | 485 | 231 | 0.0011 | 0.012 |
| GO:0055035 | plastid thylakoid membrane | 10 | 485 | 231 | 0.0011 | 0.012 |
| GO:0042651 | thylakoid membrane | 10 | 485 | 244 | 0.0016 | 0.017 |
| GO:0009570 | chloroplast stroma | 10 | 485 | 249 | 0.0019 | 0.018 |
| GO:0031977 | thylakoid lumen | 6 | 485 | 95 | 0.0019 | 0.018 |
| GO:0009532 | plastid stroma | 11 | 485 | 322 | 0.0038 | 0.034 |
|  |  |  |  |  |  |  |
|  |  |  |  |  |  |  |
| **Down-regulated GO categories** | |  |  |  |  |  |
| **GO_acc** | **Term** | **queryitem** | **querytotal** | **bgitem** | **p-value** | **FDR** |
| GO:0005739 | mitochondrion | 48 | 711 | 1276 | 0.000009 | 0.00025 |
| GO:0031980 | mitochondrial lumen | 6 | 711 | 54 | 0.00084 | 0.016 |
| GO:0005759 | mitochondrial matrix | 6 | 711 | 54 | 0.00084 | 0.016 |
| GO:0044429 | mitochondrial part | 14 | 711 | 274 | 0.001 | 0.019 |
| GO:0070469 | respiratory chain | 7 | 711 | 115 | 0.0077 | 0.13 |
